# Epidemiology of Chronic Kidney Disease, With Special Emphasis on Chronic Kidney Disease of Uncertain Etiology, in the North Central Region of Sri Lanka

**DOI:** 10.2188/jea.JE20140074

**Published:** 2015-04-05

**Authors:** Kithsiri Bandara Jayasekara, Dhammika Menike Dissanayake, Ramiah Sivakanesan, Asanga Ranasinghe, Ranawaka Hewage Karunarathna, Gardiye Waligamage Gamini Priyantha Kumara

**Affiliations:** 1 Senior Lecturer, Department of Medical Laboratory Sciences, General Sir John Kotelawala Defense University, Rathmalana, Sri Lanka; 2 Department of Pathology, Faculty of Medicine, University of Peradeniya, Peradeniya, Sri Lanka; 3 Department of Biochemistry, Faculty of Medicine, University of Peradeniya, Peradeniya, Sri Lanka; 4 Renal Unit, Provincial Director’s Office, Anuradhapura, Sri Lanka

**Keywords:** chronic kidney disease, uncertain etiology, epidemiology, North Central Province, male farmers, natural spring water

## Abstract

**Background:**

The aim of the study was to identify the epidemiology of chronic kidney disease of uncertain etiology in Sri Lanka.

**Methods:**

A cross-sectional study was carried out by analyzing health statistics, and three cohort studies were conducted (*n* = 15 630, 3996, and 2809) to analyze the demographic information, age-specific prevalence, etiology, and stage of presentation. We screened 7604 individuals for chronic kidney disease of uncertain etiology.

**Results:**

The results showed that the male:female ratio was 2.4:1, the mean age of patients was 54.7 ± 8 years, 92% of the patients were farmers, and 93% consumed water from shallow dug wells. Familial occurrence was common (36%). The prevalence of chronic kidney disease in different age groups was 3% in those aged 30–40 years; 7% in those aged 41–50 years, 20% in those aged 51–60 years, and 29% in those older than 60 years. Chronic kidney disease of uncertain etiology was diagnosed in 70.2% of patients, while 15.7% and 9.6% were due to hypertension and diabetic mellitus, respectively. The majority of patients were stage 4 (40%) at first presentation, while 31.8% were stage 3 and 24.5% were stage 5. Stage 1 and 2 presentation accounted for only 3.4%.

**Conclusions:**

Low prevalence of CKDU was noticed (1.5%) among those who consumed water from natural springs. Prevalence was highest among males, rice farming communities, and those presenting at later disease stages.

## INTRODUCTION

Chronic kidney disease (CKD) is increasingly recognized as a global public health problem. According to the World Health Organization’s Global Burden of Disease (GBD) project, kidney and urinary tract disease is the 12th most common cause of death and the 17th most common cause of disability.^1^^,^^2^ The total number of Americans living with CKD is now estimated to be 19.2 million, representing 11% of the adult population in the U.S., and 0.22% of the population is estimated to have end-stage renal disease (ESRD) resulting from CKD.^3^ According to the 2008 United States Renal Data System annual report Collins et al,^4^ the major causes of CKD leading to kidney failure in the United States are diabetes, hypertension, and glomerulonephritis, which account for 23.7 cases per million population. The study reported that 28% of the CKD patients who were greater than 65 years of age were neither diabetic nor hypertensive, suggesting that cardiovascular disease is also an important cause. Couser et al^5^ reviewed CKD in developing countries and mentioned that the percentage of CKD patients devoid of diabetes or hypertension is considerably higher in developing countries than in developed ones. Diabetes and hypertension account for 30% and 21% of ESRD patients, respectively; however, glomerulonephritis and CKD due to uncertain etiology account for a larger portion among younger patients in developing countries.^5^^,^^6^ A study involving International Society of Nephrology-sponsored screening programs in China, Mongolia, and Nepal revealed that 43% of people with CKD did not have diabetes or hypertension.^7^ The estimated prevalence of moderate CKD in developed countries is unclear but is generally thought to be between 5% and 7% of the total adult population.^8^^–^^10^

One study reported that the epidemiology of CKD in India is different from that in European and Western populations.^11^ Patients are approximately two decades younger on average, and a substantial proportion present with small kidneys and unclear CKD etiology. The study concluded that the absence of nationwide reporting systems or registries makes it difficult to determine the true incidence and prevalence.^11^

Chronic kidney disease of uncertain etiology (CKDU), a new type of nephropathy has been reported in several countries over the last few decades.^12^ The first outbreak of one type of CKDU, Balkan endemic nephropathy was reported between 1955 and 1957, initially in Serbia and soon afterwards in Croatia, Bosnia-Herzegovina, and Yugoslavia. In Yugoslavia, six foci were identified along major rivers of the Danubian basin.^13^ The prevalence rate of the disease was reported to be between 2% and 10% in the Balkan region. A striking feature of BEN was the familial occurrence of the disease; epidemiological studies have suggested that the disease is abundant in family members. Longer duration of exposure and close contact with water in affected areas have been postulated as risk factors for BEN. However, the precise etiology has still not been determined.^14^^,^^15^

CKDU has also been observed along the Pacific border of the Central American region, including Nicaragua and El Salvador, which does not appear to follow the same epidemiological patterns demonstrated in CKDU in developed countries. In a study performed in the municipality of Quezalguaque, Nicaragua in response to anecdotal reports of a high prevalence of kidney disease in agricultural regions in Northwestern Nicaragua, an overall prevalence of 12.7% for decreased eGFR (<60 mL/min/1.73 m^2^) was observed.^16^

A case-control study confirmed that the epidemiology of kidney disease in Nicaragua is different from that seen in the U.S. and other developed countries. However, Quezalguaque residents with diabetes and hypertension accounted for only a small portion of CKD.^16^ The majority of cases in this region are in young adults. Researchers have found that working in the cotton and sugar cane industries, pesticide exposure, living at lower altitude, alcoholism, and consumption of commercially produced bulk rum known as ‘Guaro Lija’ are risk factors for occurrence of the disease.^16^

CKDU, a new and predominant form of CKD in certain parts of Sri Lanka, including North Central Province (NCP), Uva Province (UP), and a few areas of the North Western Province (NWP), is threatening to reach epidemic proportions.^17^ Researchers have suggested different etiologies for CKDU in Sri Lanka. According to studies conducted in Sri Lanka, high concentration of fluoride in water and use of aluminum utensils for cooking have been suggested as risk factors.^18^ Another study revealed that CKD is distributed mainly in agricultural regions using reservoir based cascade irrigation systems. Further, the study concluded that CKD in North Central Sri Lanka was a result of chronic dietary intake of cadmium and high natural levels of fluoride in drinking water.^19^ Unrecognized environmental toxins or occupational exposures were also suggested to contribute to the increased prevalence of CKD in NCP.^20^ However, contradictory findings identified by different scientists indicated that the moderate to high levels of fluoride and cadmium in drinking water in the affected regions did not contribute to CKDU.^21^ By considering the studies which have been done so far, it is obvious that there is a need for a comprehensive study of CKDU in Sri Lanka. The epidemiology of CKDU in Sri Lanka, including emergence of the disease, stages of presentation, and age-specific prevalence of the disease, is poorly explained by the literature.

## METHODS

To identify the prevalence of CKD in each province of the country, the number of CKD patients in all districts was collected from the medical statistics division and analyzed by province. CKDU is generally seen in three provinces: NCP, the northern parts of the UP (Mahiyanganaya and Girandurukotte), and some parts of the NWP (Nikawewa). These three areas together can be treated as special geographical terrain called the North Central Region (NCR), and further information were collected only from these three provinces.^22^

A large cohort study was conducted to identify the epidemiological characteristics of CKDU in Sri Lanka. Information was collected, including basic demographic data (age, sex, occupation, and water source), from 15 630 CKD patients who attended hospitals and community renal clinics that were specifically established to follow up CKD patients in all high prevalence areas in Sri Lanka, such as Madawachchiya, Kebithigolewa, Padaviya, Padavisripura, Girandurukotte, Pollonnaruwa, Medirigiriya, Ellahera, and Nikawewa, from 2004 to 2011.

A subset of recently diagnosed CKD patients (*n* = 3996; diagnosed from 2009–2011) attending hospitals and community renal clinics in NCP were further classified into 13 age categories. Population information was collected from Sri Lanka’s census and statistics department and Anuradhapura’s Survey Department, and age-specific prevalence of the disease was calculated for the three areas that comprise the NCP. The prevalence of CKDU among CKD patients in NCP was also analyzed. According to the guidelines provided by the Ministry of Health, the diagnostic criteria used for CKDU include absence of diabetes mellitus, hypertension, urinary tract infections, or other renal diseases in the history; presence of proteinuria on two occasions; decreased eGFR (Modification of Diet in Renal Disease-MDRD formula); and presence of radiological changes, such as normal kidney in the early stage and reduction of kidney size, increased cortical echogenicity, and loss of cortico-medullary demarcation in the late stage. The earliest pathological manifestation was interstitial inflammation and interstitial fibrosis, whereas tubular atrophy and glomerulosclerosis were seen in later stages.^23^

Using the diagnostic criteria, CKDU patients (*n* = 2809) identified by health professionals in NCP (medical officers in a Renal Unit) in the above analysis were cautiously evaluated for stage of CKDU at their first clinic visit and the number of patients at each clinical stage was estimated for the period 2009 to 2011. The stage of presentation of CKDU was identified by analyzing biopsy reports and eGFR levels according to the KDOQI guidelines.^24^ We screened 7604 individuals (3832 males and 3772 females, all aged >5 years) in 14 Gramaniladhari divisions to identify proteinuria in the community in one of the high-prevalence areas of the NCP, Kebithigollaewa, using a random cluster sampling method in collaboration with medical officers in the NCP. Information on drinking water sources used by participants was collected for the preceding five years (such as natural spring, shallow dug wells, or other water sources). The urine standard dipstick method was used to detect proteinuria in early morning urine samples of the population. An individual with proteinuria on two consecutive occasions was referred to a renal clinic for further investigation of CKDU. Patients confirmed to have CKDU from the screening study and previously identified patients were also included. Prevalence of CKDU among the screened population, including previously identified patients, was analyzed according to the drinking water sources used. The study was approved by the Provincial Director of Health Services, NCP, Sri Lanka.

## RESULTS

### Contribution of CKDU among CKD community in NCP

Figure shows CKD cases reported in NCP, Northern (NP), Central (CP), Western (WP), Southern (SP), NWP, UP, Sabaragamuwa (SAP), and Eastern Province (EP) in Sri Lanka from 2000 to 2010. The highest number of CKD patients was recorded in the NCP. The mean number of CKD patients from 2000 to 2010 in NCP was 1.88 per 1000 persons, while it was only 0.40 in other provinces (Figure).

**Figure.**
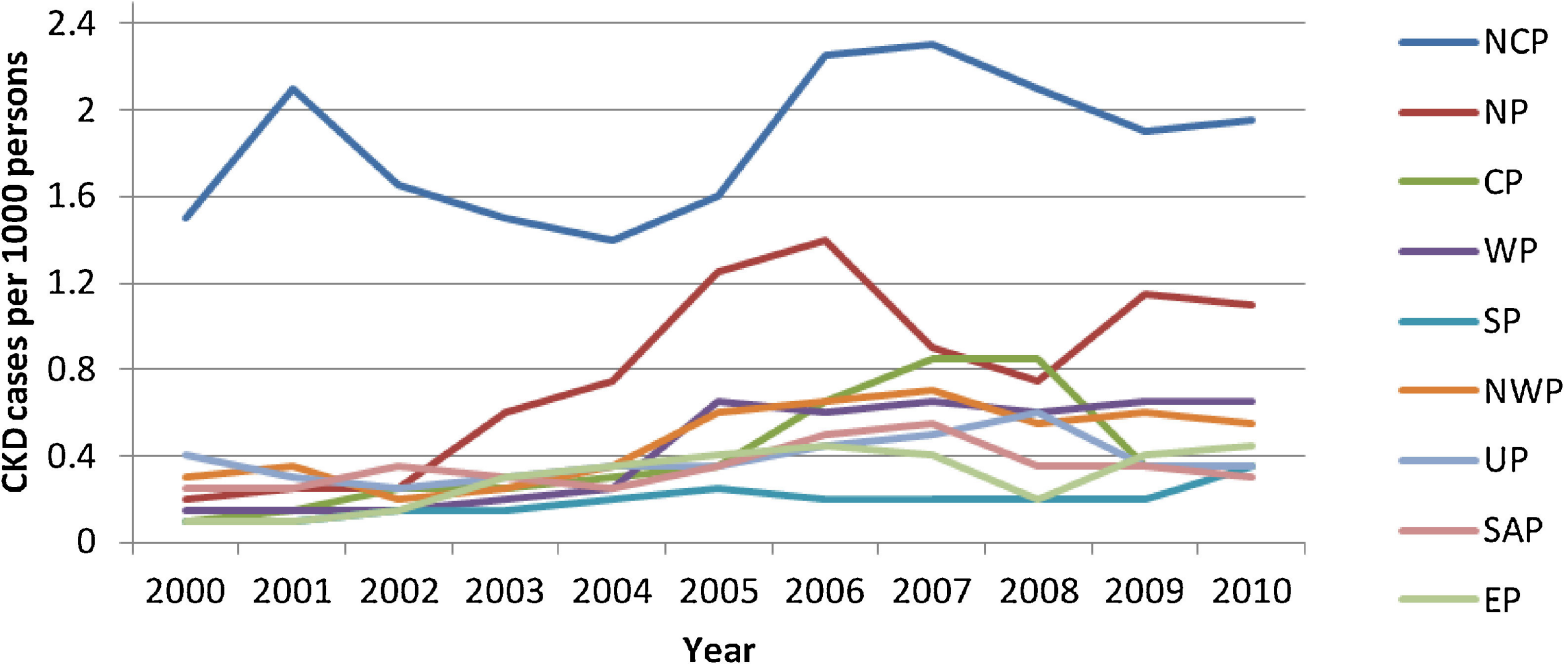
Number of chronic kidney disease patients per 1000 population in all provinces in Sri Lanka between 2000 and 2010.

### Demographic information of CKD patients in NCP

Analysis of 15 630 CKD patients revealed a male:female ratio of 2.4:1. A total of 92% of the patients were farmers, 6% were military workers, and 2% were involved in other occupations. A total of 93% of the patients consumed water from shallow dug wells for at least five years before having the disease, 6% of patients used tube wells, and only 1% consumed water directly from reservoirs.

### Age-specific CKD distribution and etiology of the disease in high-prevalence areas in NCP

The percentage of CKD patients (*n* = 3996) in the Medawachchiya, Padaviya, and Kebithigollewa areas of NCP according to age, 3% aged 30–40 years, 7% aged 41–50 years, 20% aged 51–60 years, and 29% aged >60 years. The male:female ratio was highest in the 40–44 years age group and gradually decreased with increasing age (Table 1).

**Table 1.** Age-specific prevalence of chronic kidney disease in North Central province at first diagnosis from 2009–2011

Age groups, years	Male CKD patients	Male population (% CKD)	Female CKD patients	Female population (% CKD)	Total CKD patients (%)	Male:female ratio
0–4	0	4282 (0%)	0	3978 (0%)	0 (0%)	0
5–9	6	4479 (0.1%)	5	4128 (0.1%)	11 (0.1%)	1.20
10–14	16	4126 (1%)	14	4021 (1%)	30 (0.5%)	1.14
15–19	34	4258 (1%)	24	4133 (1%)	58 (1%)	1.42
20–24	36	4968 (1%)	29	4721 (1%)	65 (1%)	1.24
25–29	40	3987 (1%)	29	3542 (1%)	69 (1%)	1.37
30–34	82	3564 (2%)	40	3561 (1%)	122 (2%)	2.05
35–39	189	3421 (5%)	45	3456 (2%)	234 (4%)	4.20
40–44	264	3129 (8%)	72	3456 (2%)	336 (5%)	3.66
45–49	381	2963 (13%)	125	2561 (5%)	506 (9%)	3.05
50–54	458	1986 (23%)	177	1689 (10%)	635 (17%)	2.58
55–59	366	1024 (35%)	134	1022 (13%)	500 (24%)	2.73
>60	949	2563 (37%)	481	2468 (20%)	1430 (29%)	2.19
Total	2821	44 750 (6%)	1175	42 736 (3%)	3996 (5%)	2.40

CKD, chronic kidney disease.

Patients were further analyzed for etiology of the disease. A total of 70% of CKD patients were identified as CKDU, while CKD was attributed to hypertension in 15.7% of patients and diabetic mellitus in 9.6% of patients (Table 2).

**Table 2.** Etiology of chronic kidney disease in North Central Province from 2009–2011

Etiology	Number of patients	Prevalence, %	Age, mean ± SD	Male:female ratio
CKDU	2809	70.2	54 ± 8	2.6:1
Hypertension	629	15.7	58 ± 12	1.6:1
Diabetic mellitus	384	9.6	56 ± 10	1.8:1
Others	174	4.4	44 ± 15	1.3:1
Total	3996	100	53 ± 12	2.4:1

CKDU, chronic kidney disease of uncertain etiology; SD, standard deviation.

### Stage of presentation

We evaluated 2809 CKDU patients to identify the stage of presentation at the first day of diagnosis. The analysis was done according to eGFR levels and biopsy reports of the patients. The majority of the CKDU patients were at stage 4 (40%), while 31.8% and 24.5% were in stages 3 and 5, respectively. Stages 1 or 2 accounted for only about 3.4% of patients. Biopsy reports showed interstitial fibrosis (IF) of any degree, absent or mild interstitial inflammation (II), tubular atrophy (TA) of any degree, with or without glomerular sclerosis (GS) in stage 1 and 2, moderate to severe IF and II and TA of any degree with or without GS in stage 3; and severe IF and II and TA in stages 4 and 5.

### Prevalence of CKDU in Kebithigollewa according to water source

In the Kebithigollewa area, 1762 individuals consumed water from natural springs and 5842 consumed water from shallow wells. A low prevalence of CKDU (1.5%) was noticed among consumers of natural spring water, and a relatively high prevalence (7.7%) was identified among consumers of water from shallow wells (Table 3).

**Table 3.** Prevalence of chronic kidney disease of uncertain etiology in the Kebithigollewa area from 2004–2011

Drinking water source	Number screened	Previously diagnosed CKDU cases	New CKDU cases	Total CKDU cases	Odds ratio (95% CI)	Total prevalence of CKDU
Males						
Natural springs	912	16	4	20	1	2%
Shallow wells	2996	286	42	328	5.48 (3.46–8.66)	11%

Females						
Natural springs	850	6	3	9	1	1%
Shallow wells	2846	103	25	128	4.40 (2.23–8.68)	4.4%

Total	7604	411	74	485		6.3%

CKDU, chronic kidney disease of uncertain etiology; CI, confidence interval.

## DISCUSSION

The present cross-sectional study was conducted in all 9 provinces of the country, and a remarkably higher number of CKD patients was noticed only in NCP. Prevalence of CKD in NCP was four times higher than in the other provinces due to the high number of CKDU patients, who comprised approximately 70% of the CKD population in NCP. A few CKDU clusters in Uva and North Western Province have also been identified^22^; however, CKDU patients in these clusters did not comprise a large percentage of total CKD patients. CKDU patients comprised only 25.6% of CKD patients in WP. Other provinces of the country were also affected with CKD, though the etiologies of the disease were primarily diabetes (30.6%) and hypertension (13.2%). It has also been reported that the difference in incidence of diabetic nephropathy between WP and other provinces was not statistically significant (*P* > 0.05).^24^

We observed a higher risk of developing CKD for male farmers engaged in agricultural activities in NCP, indicating that this group may be exposed to an unidentified etiological agent. The increasing age-specific prevalence of CKD from the age of 35 years onwards indicates the possibility of long-term exposure to an etiological agent and/or contributory factors present in the environment. The male:female ratio increased with age from the 30–34 years age group, indicating that males were more susceptible than females at younger ages; however, with continued exposure, the number of females affected also increased (Table 1). One-third of the population older than 60 years was affected with CKD in NCP. A study conducted in the U.S. in 2003 also revealed that the prevalence of CKD was 0.2% in the 20–39 years age group, 1.8% in the 40–59 years age group, and 7.6% in the 60–69 years age group.^26^ Intense farming activities and exposure to pesticides were identified as risk factors for CKDU in Nicaragua,^16^ and all of the affected CKD patients in the BEN cluster and in India’s Andhra Pradesh cluster were farmers.^27^ Moreover, the affected patients were adults between 30 and 50 years of age in both the BEN cluster,^13^ and in CKD in Central American countries.^16^

A total of 70% of CKD patients in NCP were with unknown etiology (CKDU) and only 15.7% and 9.6% were diagnosed as hypertensive and diabetic mellitus patients, respectively. However, diabetes and hypertension contributed to over 60% of the CKD cases in the WP.^25^ CKDU patients were relatively younger than patients with CKD due to hypertension and diabetes. In addition, the current study revealed that the male:female ratio of the other etiologies of CKD, such as diabetic nephropathy and hypertension, in NCP was only about 1.5:1 (Table 2), while the male:female ratio of CKDU patients was 2.6:1 in the present study and was high among CKDU patients in this region in another study.^22^

In general, the prevalence of CKD was greater in women than in men, regardless of age. A cross-sectional study reported a higher prevalence of CKD in Swiss women compared to men (4.5% in men vs 11.5% in women).^28^ Another study revealed a remarkably high prevalence in both men and women but noted that women had a tendency to have a higher prevalence of CKD than men (14.4% in men vs 16.2% in women, *P* < 0.01).^29^ According to an Australian diabetes study, statistically significant gender differences exist in the prevalence of CKD among diabetic patients (9.3% in men vs 13.0% in women, *P* = 0.002).^30^ Further, higher prevalence of CKD in women compared to men has been observed across age categories and also in various ethnic groups.^31^

The majority of the CKDU patients were in stage 4 (40%) in the present study, with 31.8% in stage 3 and 24.5% in stage 5. However, stage 1 or 2 disease accounted for only 3.4%. Two-parameter standard urine dipstick method is a widely used screening method to identify CKD patients in community settings. However, the method is not sensitive enough to detect patients with stage 1 and 2 (early) CKD. A histopathological study showed that around 50% of patients were in stage 3, while 27% of CKDU patients were asymptomatic.^23^

We studied over 7000 individuals (one-third of the population in this area) in the high-CKDU-prevalence area of Kebithigollewa, where 23% of people consumed water from natural springs and 77% from shallow dug wells. The area contains 18 natural springs, and people who consumed water from springs were less affected than people who consumed water from shallow dug wells in Kebithigollewa (Table 3). Therefore, a special health education program needs to be conducted to encourage use of spring water at least for drinking and cooking. A total of 92% of CKD patients in NCR consumed water from shallow dug wells; therefore, it can be speculated that the etiology of CKDU is strongly related to drinking water sources. Observations in Andhra Pradesh in India suggest that some elements present in ground water (fluoride, calcium, magnesium, and sulfate) may be contributing factors for CKDU.^31^
